# DeepLION: Deep Multi-Instance Learning Improves the Prediction of Cancer-Associated T Cell Receptors for Accurate Cancer Detection

**DOI:** 10.3389/fgene.2022.860510

**Published:** 2022-04-11

**Authors:** Ying Xu, Xinyang Qian, Xuanping Zhang, Xin Lai, Yuqian Liu, Jiayin Wang

**Affiliations:** ^1^ Department of Computer Science and Technology, School of Electronic and Information Engineering, Xi’an Jiaotong University, Xi’an, China; ^2^ Institute of Data Science and Information Quality, Shaanxi Engineering Research Center of Medical and Health Big Data, Xi’an Jiaotong University, Xi’an, China

**Keywords:** T cell receptor, TCR repertoire data analysis, cancer-associated TCR, machine learning approach, multi-instance learning, deep learning framework

## Abstract

Recent studies highlight the potential of T cell receptor (TCR) repertoires in accurately detecting cancers *via* noninvasive sampling. Unfortunately, due to the complicated associations among cancer antigens and the possible induced T cell responses, currently, the practical strategy for identifying cancer-associated TCRs is the computational prediction based on TCR repertoire data. Several state-of-the-art methods were proposed in recent year or two; however, the prediction algorithms were still weakened by two major issues. To facilitate the computational processes, the algorithms prefer to decompose the original TCR sequences into length-fixed amino acid fragments, while the first dilemma comes as the lengths of cancer-associated motifs are suggested to be various. Moreover, the correlations among TCRs in the same repertoire should be further considered, which are often ignored by the existing methods. We here developed a deep multi-instance learning method, named DeepLION, to improve the prediction of cancer-associated TCRs by considering these issues. First, DeepLION introduced a deep learning framework with alternative convolution filters and 1-max pooling operations to handle the amino acid fragments with different lengths. Then, the multi-instance learning framework modeled the TCR correlations and assigned adjusted weights for each TCR sequence during the predicting process. To validate the performance of DeepLION, we conducted a series of experiments on several cohorts of patients from nine cancer types. Compared to the existing methods, DeepLION achieved, on most of the cohorts, higher prediction accuracies, sensitivities, specificities, and areas under the curve (AUCs), where the AUC reached notably 0.97 and 0.90 for thyroid and lung cancer cohorts, respectively. Thus, DeepLION may further support the detection of cancers from TCR repertoire data. DeepLION is publicly available on GitHub, at https://github.com/Bioinformatics7181/DeepLION, for academic usage only.

## Introduction

T cells that respond to tumor antigens are the central mediators of cancer immunity (Gubin et al., 2014; Tran et al., 2014; Tumeh et al., 2014). For a cancer patient, the T cell repertoire often undergoes some cancer-specific changes during the tumor progression (Schreiber et al., 2011), where whose T cell receptors (TCRs) are defined as cancer-associated TCRs (caTCRs). It is reported that some caTCRs may share universal biochemical signatures (Chowell et al., 2015; Li et al., 2016). Recent studies further indicated that there are shared antigens and TCRs among the patients with the same cancer type or subtype (Kvistborg et al., 2013; Dhodapkar and Dhodapkar, 2016). The rapid development of immune repertoire sequencing (IR-seq) (Kirsch et al., 2015) enables a comprehensive view of TCR repertoires on both individual and population levels. Then, it is natural that several computational frameworks were proposed to predict the caTCRs, some of which further attempted to distinguish the cancer-associated repertoires from those non-cancer ones.

However, accurately predicting the caTCRs is quite challenging work, mainly due to the tremendous heterogeneity on personal antigen landscapes, while the lack of knowledge about the cancer antigens inducing spontaneous T cell responses brings additional difficulty (Coulie et al., 2014). To complete this work, several studies attempted to mine the biochemical properties of caTCRs from TCR sequencing (TCR-seq) data. The majority of them focused on the TCRβ chain complementarity determining region 3 (CDR3) because it primarily determines the antigen-binding specificity as the somatically generated portion of the gene. Given the computational difficulty of analyzing the entire CDR3 sequences, some approaches simplified computational processes by preprocessing the original sequences into length-fixed overlapping adjacent amino acid (AA) fragments and predicted the caTCRs by identifying key motifs in the fragments, but there is currently no consensus on the sequence decomposition strategy. Cinelli et al. decomposed the sequences into triplets (denoted as 3-mers) and then selected the key motifs from the 3-mers with 1-dimensional (1-D) Bayesian classifier to train their support vector machine model for repertoire classification (Cinelli et al., 2017). Sun et al. adopted Cinelli’s sequence decomposition strategy and trained their LPBoost model with the frequencies of 3-mers to identify key motifs and classify repertoires (Sun et al., 2017). Interestingly, Ostmeyer’s study partitioned the sequence into 4-mers (other than 3-mers) based on the analysis of the X-ray crystal structure of human TCR bound to peptide-major histocompatibility complex (MHC) before distinguishing tumor tissue from adjacent healthy tissue in colorectal and breast cancer samples (Ostmeyer et al., 2019). Furthermore, the X-ray crystal structure analysis (Ostmeyer et al., 2019) revealed that the size of adjacent CDR3 residues in direct contact with peptide varied from two to eight, implying that the length of the key biochemical motifs in TCRs should not be fixed. Therefore, decomposing sequences into *z*-mers, which limits these approaches to identifying length-fixed key motifs, is considered to lead to information loss and may harm subsequent model classification.

In contrast to the above approaches, some studies investigated the entire CDR3 sequences to consider the correlations among sequences. Emerson et al. built a statistical classification framework that could predict cytomegalovirus (CMV) status from the resulting catalog of CDR3 sequences (Emerson et al., 2017). Yokota’s approach compared the TCR repertoires in low dimensions based on entire sequence information, which estimated the low-dimensional structure after embedding the pairwise high-dimensional sequence dissimilarities (Yokota et al., 2017). Both of these approaches concentrated on the similarity comparisons among the entire sequences. However, only partial residuals of TCR contribute to antigen-binding specificity (Ostmeyer et al., 2019), the sequence comparison approaches were unable to focus on these residuals, potentially resulting in poor performances. DeepCAT is a deep learning framework enabling *de novo* prediction of caTCRs (Beshnova et al., 2020). Antigen-specific sequences in each repertoire were selected to be predicted by a set of the trained convolutional neural network (CNN) models after clustering CDR3 sequences based on their similarity (Zhang et al., 2020). The cancer score, which quantifies the likelihood that a repertoire is associated with cancer, was calculated using the average of the sequence predictions. Compared to the above two approaches, DeepCAT was able to predict the caTCRs more accurately and performed better in repertoire classification due to the cluster analyses in data preprocessing and deep learning’s excellent feature extraction ability. However, DeepCAT ignored the correlations among TCRs in the same repertoire for the simple definition of cancer score, which assigned the same weight for all TCRs in a repertoire whereas they may own distinct weights. Ostmeyer’s approach (Ostmeyer et al., 2019) attempted to model the TCR correlations with multi-instance learning (MIL), but it used the standard MIL assumption (Dietterich et al., 1997; Foulds and Frank, 2010), predicting the repertoire as cancerous based on the presence of only one abnormal TCR, which is unsuitable and has a risk of increased false positives because a cancer patient’s repertoire typically contains many caTCRs that are related to one another.

**FIGURE 1:**
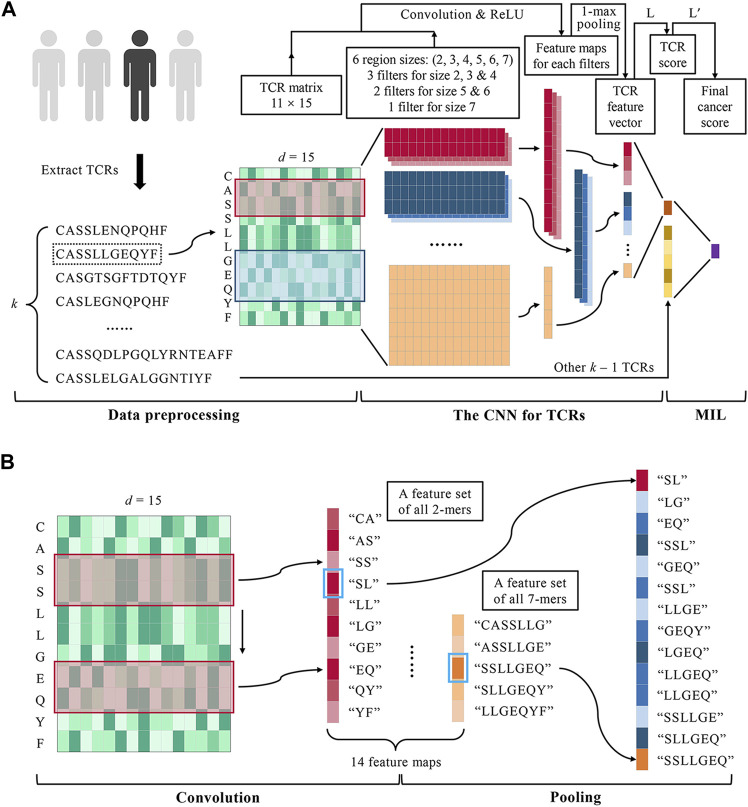
DeepLION for accurate TCR repertoire prediction. **(A)** The workflow of DeepLION is divided into three parts: data preprocessing, the CNN for TCRs, and MIL. During data preprocessing, the top *k* most abundant TCR sequences were extracted from each repertoire after removing unqualified sequences and they were encoded into matrixes by the Beshnova matrix. The CNN for TCRs consisted of 14 convolution filters covering six various region sizes, 1-max pooling operations, and a one-layer linear classifier L. The TCR matrixes were input to the CNN and their scores were output. In the MIL part, DeepLION employed another one-layer linear classifier L′ to aggregate *k* TCR scores to predict the repertoire. **(B)** The details of the convolution and pooling operations of CNN in DeepLION. When a 2 × *d* convolution filter (the red box) performed a complete convolution operation on the TCR matrix from top to bottom, it could be regarded as extracting the biochemical features of the 2-mers such as "CA", "AS", etc., and then a 10 × 1 feature map, a feature set of all 2-mers, was generated. Other filters performed similar convolution operations and 14 feature maps were obtained. The maximum value of each map (marked with a blue box) was selected by a 1-max pooling operation, which could be viewed as the feature of the *z*-mers most likely to be the cancer-specific motif. These features were interconnected to generate a 14 × 1 TCR feature vector.

In summary, there is a dearth of caTCR prediction approaches that take into account the cancer-associated biochemical motifs with various lengths and correctly model the correlations among TCRs in the same repertoire. To bridge this gap, we developed a deep MIL method called DeepLION in this study to improve the prediction of caTCRs using TCR-seq data (Figure 1). On one hand, the CNN with alternative convolution filters and 1-max pooling operations was used to handle AA fragments with different lengths in TCRs, where various lengths of cancer-associated motifs were identified; on the other hand, the MIL part of DeepLION assigned appropriate weights for each TCR after modeling the TCR correlations during the predicting process. We evaluated the performance of DeepLION on several cohorts of patients from nine cancer types and found that it achieved higher prediction accuracies, sensitivities, specificities, and areas under the receiver operating characteristic (ROC) curve (AUCs) for most of the cohorts compared with the current state-of-the-art methods, with the AUCs for thyroid and lung cancer cohorts reaching 0.97 and 0.90, respectively. Thus, DeepLION can accurately predict the caTCRs and distinguish the cancer-associated repertoires from those non-cancer ones, potentially assisting in the detection of malignancies based on TCR repertoire data.

## Materials and Methods

DeepLION is a deep MIL method based on TCR-seq data for caTCR prediction. The workflow of DeepLION consisted of three parts: data preprocessing, the CNN for TCRs, and MIL (Figure 1A). First, *k* TCRβ CDR3 sequences with the highest abundance in the repertoire were selected and encoded into the matrixes using AA biochemical features. After data preprocessing, the TCR matrixes with different sizes were fed into a deep CNN model, where the biochemical features of key motifs with various lengths were extracted by alternative convolution filters and 1-max pooling operations, and then the scores of TCRs, the probabilities that they were caTCRs, were calculated. Finally, a one-layer linear classifier L′ was employed to aggregate *k* TCR scores as the cancer score of the repertoire, which is used to predict whether it is cancerous.

### Data Preprocessing

We collected the CDR3 of TCRβ from TCR-seq data to study. Considering that low-quality CDR3 sequences will affect the downstream analysis, the following types of sequences were removed as described in the previous study (Beshnova et al., 2020): *1*) too short (<10) or too long (>24) sequences; *2*) sequences containing special characters (X, +, ∗, etc.); *3*) incomplete sequences, according to the ImMunoGeneTics (IMGT) nomenclature (Lefranc et al., 2015), not starting with the cysteine (C) or not ending with the phenylalanine (F); and *4*) sequences where variable gene locus was not solved. There were some overlapping TCR sequences between healthy individuals and cancer patients, which were considered irrelevant to cancer and therefore also needed to be excluded. We used known training data to generate a reference dataset (denoted as D_R_) containing sequences that appeared at a high frequency in both healthy individuals and cancer patients (the top 20,000 sequences with the highest cloning frequency in each TCR-seq sample). Any sequences in each sample appearing in the D_R_ were removed. After the above sequences were removed, the remaining TCR sequences were sorted in descending order of cloning frequency and the top *k* sequences were extracted for downstream analysis.

The raw TCR sequences were not directly input to the CNN because their antigen-binding ability was not well represented. AAs in TCR sequences can be represented by biochemical features, and a TCR sequence with length *l* is able to be encoded by a 20 × *d* feature matrix for 20 AAs into an *l* × *d* TCR matrix. The AA index database (Kawashima and Kanehisa, 2000) documented 566 AA indices containing rich biochemical information based on previous literature. Since the original 566 AA indices are very large, directly utilizing them to characterize AAs may lead to a large input data size and too many parameters of CNN, which may cause problems such as high computational complexity and overfitting. In addition, many of the original AA indices are highly correlated with each other. As a result, we considered dimensionality reduction of the original AA indices. Currently, many studies have extracted low-dimensional orthogonal features from high-dimensional AA indices, reducing the dimensionality of a large number of AA indices with minimal information loss. Kidera et al. derived a 20 × 10 feature matrix from 188 AA indices (Kidera et al., 1985), and Atchley et al. derived a 20 × 5 feature matrix from 494 AA indices (Atchley et al., 2005). Beshnova et al. employed principal component analysis (PCA) to generate a 20 × 15 feature matrix from 531 AA indices to characterize AAs (Beshnova et al., 2020). Considering that the Beshnova matrix was obtained from the largest number of AA indices and encompassed the most biochemical information (explaining more than 95% of the variation in the data), we used the Beshnova matrix in our experiments (*d* = 15) to encode the TCR sequences into matrixes.

### Identifying Various Lengths of Cancer-Associated Motifs

The CNN is able to predict whether a TCR sequence is associated with cancer. TextCNN in natural language processing (NLP) firstly applied the CNN model to sequence analysis (Zhang and Wallace, 2015). Referring to the idea of TextCNN, the model consisted of a convolutional layer, a pooling layer, and a linear layer, as shown in Figure 1A. Different from TextCNN, we developed innovative convolution filters in the convolutional layer to handle the amino acid fragments with different lengths according to the X-ray crystal structure analysis (Ostmeyer et al., 2019). For a TCR matrix with dimension *l* × *d*, the model extracted its features by a set of convolution filters with various sizes respectively, performed 1-max pooling operations for the low-dimensional feature maps obtained by each convolution filter, and concatenated the pooled results to generate a TCR feature vector. The TCR score, the probability of being a caTCR, was obtained by a one-layer linear classifier finally.

The convolutional layer of the model was designed with multiple convolution filters to extract the key biochemical motifs with distinct lengths in TCRs. By analyzing the X-ray crystal structure, Ostmeyer et al. identified the CDR3 residues in contact with peptide, which were thought to make the greatest contribution to the antigen-binding specificity of the TCRs, and determined the length of the AA fragments according to the analysis (Ostmeyer et al., 2019). We counted the contiguous CDR3 residue regions (size ≥2) in the 55 CDR3 sequences used for analysis (Table 1) and observed that the regions ranging in size from 2 to 8 were present, with regions of sizes 2–4 occurring more frequently and regions of size eight occurring least frequently at 0.017. These contiguous regions were considered as potential cancer-specific motifs that contribute to the antigen-binding ability of TCRs. After excluding the regions with low frequencies (<0.05), we designed a set of various convolution filters and specified the number of corresponding convolution filters according to the occurrence frequencies of the regions (Table 1). As shown in Table 1 and Figure 1A, the convolution filters with six different sizes were designed to extract features from TCR matrixes, and the number of convolution filters with each size was positively correlated with the occurrence frequency of the corresponding region, for a total of 14 convolution filters. Every complete convolution operation is defined as:

$$\begin{array}{c} {𝓸}_{a}^{i,F_{j}}=\sigma \left(W^{F_{j}}\cdot M_{i}\left[a:a+h-1\right]+b^{F_{j}}\right)\in {𝓸}^{i,F_{j}}={\left[{𝓸}_{1}^{i,F_{j}},\ldots ,{𝓸}_{l-h+1}^{i,F_{j}}\right]}^{\text{T}}, \end{array}$$ (1)

where the output sequence ${𝓸}^{i,F_{j}}\in {\mathbb{R}}^{l-h+1}$ is composed of the results of each convolution ${𝓸}_{a}^{i,F_{j}}\left(a=1,\ldots ,l-h+1\right)$ , the activation function σ(x)=max(0, x) is the Rectified Linear Unit (ReLU), $W^{F_{j}}\in {\mathbb{R}}^{h\times d}$ and $b^{F_{j}}\in \mathbb{R}$ are respectively the weight matrix and bias of the *j*th convolution filter $F_{j}$ with size h×d , $M_{i}\in {\mathbb{R}}^{l\times d}$ is the *i*th TCR matrix, $M_{i}\left[a:b\right]$ denotes the submatrix from row *a* to row *b* in $M_{i}$ , and ∙ denotes the dot product (a sum of element-wise multiplications) between the submatrix and the filter. Equation 1 showed the convolution operation that one convolution filter performed on the TCR matrix, and then the corresponding feature map, $o^{i,F_{j}}$ , was obtained. Taking the 11 × *d* TCR matrix shown in Figure 1B as an example, when the convolution filter with size 2 × *d* performed a complete convolution operation on the matrix from top to bottom, it could be regarded as extracting the biochemical features of the 2-mers in TCRs such as "CA", "AS", etc., and finally obtaining the 10 × 1 feature map, the feature set of all 2-mers, which probably included cancer-specific motifs. The filters with other sizes performed similar convolution operations. Because the frequencies of key motifs with different lengths varied, the numbers of convolution filters with various sizes were adjusted to give them appropriate weights in the model. ReLU was chosen as the activation function following the convolution operations due to its low computational complexity. Additionally, it can avoid the vanishing gradient or exploding gradient problems that Sigmoid and Tanh can cause. And the disadvantage of ReLU, the dead ReLU problem, was mitigated by using the Xavier initialization (Glorot and Bengio, 2010).

**TABLE 1:** The situation of continuous CDR3 residue regions and convolution filter design.

Size of region	Number of region	Frequency of region	Size of filter	Number of filter
2	12	0.207	2 × *d*	3
3	12	0.207	3 × *d*	3
4	13	0.224	4 × *d*	3
5	8	0.138	5 × *d*	2
6	7	0.121	6 × *d*	2
7	5	0.086	7 × *d*	1
8	1	0.017	—	—

The 1-max pooling function was adopted to pool the feature maps generated after convolution operations of each convolution filter, reducing the mapping dimension to 1. The following pooling functions are commonly used: *1*) the element-wise maximum function ( $f_{\text{Max}}$ ); *2*) the element-wise average function ( $f_{\text{Avg}}$ ); *3*) the log-sum-exp (LSE) function ( $f_{\text{LSE}}$ ) (Sahasrabudhe et al., 2021). These are defined as:

$$\begin{array}{c} f_{\text{Max}}\left({𝓸}^{i,F_{j}}\right)=\max\left(\left\{{𝓸}_{1}^{i,F_{j}},\ldots ,{𝓸}_{l-h+1}^{i,F_{j}}\right\}\right), \end{array}$$ (2)

$$\begin{array}{c} f_{\text{Avg}}\left({𝓸}^{i,F_{j}}\right)=\frac{1}{l-h+1}\overset{l-h+1}{\underset{a=1}{\sum }}{𝓸}_{a}^{i,F_{j}},\;\text{and} \end{array}$$ (3)

$$\begin{array}{c} f_{\text{LSE}}\left({𝓸}^{i,F_{j}}\right)=\log\left(\frac{1}{l-h+1}\overset{l-h+1}{\underset{a=1}{\sum }}\exp\left({𝓸}_{a}^{i,F_{j}}\right)\right), \end{array}$$ (4)

where ${𝓸}^{i,F_{j}}\in {\mathbb{R}}^{l-h+1}$ is the output sequence of $F_{j}$ with size h×d after the convolution operation on $M_{i}$ . The feature maps obtained could be viewed as the feature sets of all *z*-mers (z=2,…,7) . Regarding that the goal of CNN was distilling the potential cancer-specific motifs in TCRs, we focused on the most contributing features in the feature sets, which are most likely from the key motifs, and ignored the features of other *z*-mers. With the 1-max pooling function, the most contributing features of each feature set were extracted and other non-key motifs’ features were discarded, whereas the features pooled by the average function and the LSE function were affected by other non-key motifs, which caused adverse effects on the classification ability of the model. Considering that one TCR may contain multiple short key motifs with the same length, the drawback that the 1-max pooling operation can only extract the feature of one key motif from one sequence can be compensated by the design of multiple convolution filters with the same size in the convolutional layer. As shown in Figure 1B, the most contributing features (marked with blue boxes) extracted by pooling were interconnected to generate a 14 × 1 TCR feature vector as:

$$\begin{array}{c} p^{i,F_{j}}=f_{\text{Max}}\left({𝓸}^{i,F_{j}}\right)\in p^{i}={\left[p^{i,F_{1}},\;\ldots ,p^{i,\;\;F_{14}}\right]}^{\text{T}}. \end{array}$$ (5)

Equation 5 showed the 1-max pooling operation performed on the feature map, and then the TCR feature vector, $p^{i}$ , was obtained, which consisted of the most contributing features from the feature maps.

Ultimately, a one-layer linear classifier L was applied to aggregate the features extracted from each convolution kernel and predict the score for that TCR sequence. L that assigned scores to the TCRs is given by:

$$\begin{array}{c} {\tilde{y}}_{i}=P\left(y_{i}=1|M_{i}\right)=\sigma \prime \left(W^{L^{T}}p^{i}+b^{\text{L}}\right), \end{array}$$ (6)

where $P\left(y_{i}=1|M_{i}\right)$ denotes the probability that the TCR is associated with cancer, the activation function σ′(x)=1/(1+exp(−x)) is the sigmoid function to normalize the scores, and $W^{\text{L}}\in {\mathbb{R}}^{14}$ and $b^{\text{L}}\in \mathbb{R}$ are respectively the weight matrix and bias of L. Equation 6 showed the operation process in L, and the probability that the TCR was associated with cancer was obtained. The TCR was predicted to be caTCR when ${\tilde{y}}_{i}>0.5$ , and was otherwise predicted to be noncancerous. A multi-layer linear classifier is capable of fitting the data better, but it also makes the structure of CNN more complicated and introduces the risk of overfitting. To reduce overfitting, a one-layer linear classifier is applied to the model to predict the TCR scores.

The CNN model jointly learns the various convolution filters and L so that it is end-to-end trainable and the preprocessed TCRs and the corresponding labels are needed for model training. Because the probability that a TCR is a caTCR obeys a Bernoulli distribution, the log-likelihood function (also known as the cross-entropy function) was used as the loss function to train the model, which is defined as:

$$\begin{array}{c} {ℒ}_{\text{CNN}}=-\left[{\tilde{y}}_{i}\text{ln}{\tilde{y}}_{i}+\left(1-{\tilde{y}}_{i}\right)\text{ln}\left(1-{\tilde{y}}_{i}\right)\right]. \end{array}$$ (7)

During the training process, random dropouts at a rate of 40% were applied to L to mitigate overfitting.

Because zero-padding will alter the distribution of input data, DeepCAT (Beshnova et al., 2020) built five distinct CNN models for TCR sequences from 12 to 16 in length, which made itself cumbersome and unable to utilize the information of TCRs with other lengths, whereas TCRs with different lengths were processed by one model in this method. In the actual training process, we added zeros to the end of the shorter sequences to achieve the length of the longest sequences (*l* = 24) to ensure the consistency of the dimensionality of the input TCR matrixes. Because the features of all motifs containing zero viewed as non-key motifs were discarded after the 1-max pooling operations in the model, the classification ability of the model did not deteriorate.

### Multi-Instance Learning Modeling the TCR Correlations

Predicting whether a repertoire is cancer-associated from the TCRs in every repertoire can be described as MIL, where the TCRs are instances and the repertoires are bags. The standard MIL assumption assumes that each instance in a bag can be classified as either positive (1) or negative (0), and the label of a bag is 1 when including one or more positive instances (Dietterich et al., 1997; Foulds and Frank, 2010). Ostmeyer et al. applied this assumption in their study (Ostmeyer et al., 2019). However, it is inappropriate to predict the repertoire as having cancer by the presence of a non-normal TCR because a cancer patient usually contains many caTCRs, which are somehow related to each other. In addition, the needed labels of TCRs are unknown, which means that it is difficult to know whether a TCR is associated with cancer or not. Although Beshnova et al. obtained potential caTCRs used for model training from TCGA tumor RNA-seq samples in advance in their study by TRUST (Li et al., 2017) and sequence filtering based on a reference database (Beshnova et al., 2020), the evidence that all sequences were positive was lacking. The definition of cancer score for the repertoire by averaging the predictions for TCRs in a repertoire was also inaccurate because we could not prove that all TCRs enjoyed the same weight. Therefore, we designed a one-layer linear classifier L′ as an aggregating function to combine the scores of *k* TCRs collected from repertoires to predict the repertoire. L′ is defined as:

$$\begin{array}{c} \tilde{Y}=P\left(Y=1|\left\{M_{1},\ldots ,M_{k}\right\}\right)=\sigma \prime \left(W^{L\prime T}{\left[{\tilde{y}}_{1},\ldots ,{\tilde{y}}_{k}\right]}^{\text{T}}+b^{\text{L}\prime }\right), \end{array}$$ (8)

where $P\left(Y=1|\left\{M_{1},\ldots ,M_{k}\right\}\right)$ denotes the probability that the repertoire has cancer, the activation function σ′(x) is the sigmoid function, and $W^{L\prime }\in {\mathbb{R}}^{k}$ and $b^{{\text{L}}^{\text{′}}}\in \mathbb{R}$ are respectively the weight matrix and bias of L′ . Equation 8 showed the operation process in L′ , which was similar to Eq. 6, and the probability that the repertoire was associated with cancer was obtained. The repertoire was predicted to be cancer-associated when $\tilde{Y}>0.5$ , and was otherwise predicted to be noncancerous. A MIL model consisting of the CNN and L′ is also end-to-end trainable, whose loss function is the log-likelihood function defined as:

$$\begin{array}{c} {ℒ}_{\text{MIL}}=-\left[\tilde{Y}\text{ln}\tilde{Y}+\left(1-\tilde{Y}\right)\text{ln}\left(1-\tilde{Y}\right)\right]. \end{array}$$ (9)

Instead of simply taking the max value (Ostmeyer et al., 2019) or the average (Beshnova et al., 2020) value of all TCR scores as the cancer score of the repertoire, L′ was capable of learning the interrelationships among TCRs and assigning the appropriate weights to each TCR after model training. Similar to the idea of L, the multi-layer linear classifier was replaced by L′ and random dropouts at a rate of 40% were applied to L′ during training in order to alleviate overfitting.

## Results

We conducted a series of experiments on several cohorts of patients covering multiple cancers and healthy donors to validate the performance of DeepLION. In section 3.1, we detailed how we acquired the data for the experiments. In section 3.2, we assessed the capacity of the CNN framework in DeepLION to predict the caTCRs. In section 3.3, we evaluated the performance of the entire DeepLION when predicting repertoires.

### Collecting the Data

We used the publicly available TCR-seq data from Adaptive Biotechnologies immuneACCESS online database (IA), which contains eight groups of peripheral blood mononuclear cell (PBMC) samples with diverse cancer types and one group of non-cancer PBMC samples. To validate the performance of the models on Asian patients, the TCR-seq data from the clinical database of Geneplus Technology Ltd. in Shenzhen (Geneplus) were also used (Lan et al., 2020; Li et al., 2021), which included PBMC and tumor-infiltrating T lymphocyte (TIL) samples from patients with thyroid cancer (THCA) and lung cancer, as well as non-cancer PBMC samples. Table 2 shows the specifics of the various datasets that were used in the experiments.

**TABLE 2:** The specifics of the datasets.

Source	Disease	Cell type	Data type	Sample size
IA	Melanoma	PBMCs	TCR-seq	21
	BRCA	PBMCs	TCR-seq	16
	Ovarian	PBMCs	TCR-seq	4
	Pancreatic	PBMCs	TCR-seq	7
	Bladder	PBMCs	TCR-seq	30
	GBM	PBMCs	TCR-seq	15
	Lung	PBMCs	TCR-seq	29
	CRC	PBMCs	TCR-seq	3
	Non-cancer	PBMCs	TCR-seq	786
Geneplus	THCA	PBMCs and TILs	TCR-seq	170
	Lung	PBMCs and TILs	TCR-seq	184
	Non-cancer	PBMCs	TCR-seq	260

IA, Adaptive Biotechnologies immuneACCESS online database; BRCA, breast cancer; GBM, glioblastoma multiforme; CRC, colorectal cancer; THCA, thyroid cancer; PBMCs, peripheral blood mononuclear cells; TILs, tumor-infiltrating T lymphocytes; TCR-seq, T cell receptor-sequencing.

In section 3.2, we gathered the training data of DeepCAT to train the CNN framework in DeepLION. The label-encoded training data was composed of cancer (*n* = 30,000) and non-cancer (*n* ∼ 60,000) TCR CDR3 sequences. The cancer sequences were derived from The Cancer Genome Atlas (TCGA) (Tomczak et al., 2015) tumor RNA-sequencing (RNA-seq) samples covering multiple cancers using TRUST algorithm (Li et al., 2017), whereas non-cancer sequences were derived from the healthy individuals H_2_ (*n* = 120) (Emerson et al., 2017), which are independent of healthy individuals H_1_ (*n* = 666). The test data consisted of two datasets (T_1_ and T_2_). T_1_ consisted of eight groups of samples, each of which contained cancer and H_1_ PBMC samples from IA. All TCR-seq data in T_2_ were obtained from Geneplus, which were collected from Asian populations. T_2_ was composed of two groups of samples: the first group contained THCA PBMC & TIL samples (*n* = 170) and the PBMC samples from the healthy individuals H_3_ (*n* = 260), and the second group contained lung cancer PBMC and TIL samples (*n* = 184) and H_3_ PBMC samples. Table 3 shows the training and test data in section 3.2.

**TABLE 3:** The training and test data in experiments.

Section	Data type	Data source	Disease	Cell type	Sample size
3.2	Training data	TCGA	Multiple cancers	—	30,000
		IA	Non-cancer (H_2_)	—	∼60,000
	Test data	IA (T_1_)	Melanoma	PBMCs	21
			BRCA	PBMCs	16
			Ovarian	PBMCs	4
			Pancreatic	PBMCs	7
			Bladder	PBMCs	30
			GBM	PBMCs	15
			Lung	PBMCs	29
			CRC	PBMCs	3
			Non-cancer (H_1_)	PBMCs	666
		Geneplus (T_2_)	THCA	PBMCs and TILs	170
			Lung	PBMCs and TILs	184
			Non-cancer (H_3_)	PBMCs	260
3.3	Training and test data	Geneplus (T_2_)	THCA	PBMCs and TILs	170
			Lung	PBMCs and TILs	184
			Non-cancer (H_3_)	PBMCs	260

TCGA, The Cancer Genome Atlas; IA, Adaptive Biotechnologies immuneACCESS online database; BRCA, breast cancer; GBM, glioblastoma multiforme; CRC, colorectal cancer; THCA, thyroid cancer; PBMCs, peripheral blood mononuclear cells; TILs, tumor-infiltrating T lymphocytes.

In section 3.3, we used T_2_ to train and test the entire DeepLION due to the larger sizes of cancer samples in T_2_ (Table 3). The samples in each group were randomly divided into five equal parts, three of which were used as the training set, one as the validation set, and one as the test set.

### Predictive Capacity Evaluation of the CNN Framework in DeepLION for TCRs

DeepCAT (M^CAT^) (Beshnova et al., 2020) is currently a preferred method for *de novo* caTCR prediction from the peripheral blood. To validate the performance of the CNN framework in DeepLION (M^CNN^) when predicting the caTCRs, we conducted experiments to compare M^CNN^ with M^CAT^. M^CAT^ and M^CNN^ shared the same training data (Table 3), and we processed the data as described in M^CAT^.

Before the training process, all the training sequences were encoded into *l* × 15 TCR matrixes (*d* = 15) by the Beshnova matrix (Beshnova et al., 2020). To gain the final model, we trained M^CNN^ five times independently. In each training process, we randomly selected two-thirds of the training data (20,000 cancer and 40,000 non-cancer samples) as the training set and used them to train the model for 1,000 epochs at a learning rate of 0.001 with the assurance that the model reached convergence. The remaining training data were used as the validation set and AUCs were estimated to evaluate the trained models. Ultimately, the five trained models had similar AUCs, and the model with the highest AUC (0.85) was selected as the final model. The trained M^CAT^ was obtained from Github.

All the test data (Table 3) were processed in several steps. First, the top 10,000 most abundant sequences (*k =* 10,000) of each sample were extracted after low-quality sequences were removed. Second, we used iSMART (Zhang et al., 2020) with default parameters to cluster the sequences, and then the antigen-specific sequences were selected. Third, all the sequences were encoded into TCR matrixes for the downstream analysis.

The trained M^CAT^, as well as the trained M^CNN^, was applied to the processed test data, and the cancer score of each sample was defined by averaging all the input sequence scores in the sample. The sensitivities (at 0.9 specificities) and AUCs of both models were estimated for evaluating the models (Table 4). The results showed that M^CNN^ performed significantly better than M^CAT^ in terms of both the sensitivities and AUCs on each group of samples except for the melanoma, ovarian cancer, and colorectal cancer samples in T_1_, where M^CNN^’s performance was close to that of M^CAT^.

**TABLE 4:** The performance of models on different cancer samples.

	T_1_ ^a^								T_2_	
	Melanoma (21)		BRCA (16)		Ovarian (4)		Pancreatic (7)		THCA (170)	
	M^CAT^ ^b^	M^CNN^	M^CAT^	M^CNN^	M^CAT^	M^CNN^	M^CAT^	M^CNN^	M^CAT^	M^CNN^
SEN	0.762	0.762	0.438	0.750	1	1	0.714	1	0.353	0.453
AUC	0.912	0.900	0.854	0.892	0.988	0.989	0.945	0.962	0.692	0.724
	**Bladder (30)**		**GBM (15)**		**Lung (29)**		**CRC (3)**		**Lung (184)**	
	M^CAT^	M^CNN^	M^CAT^	M^CNN^	M^CAT^	M^CNN^	M^CAT^	M^CNN^	M^CAT^	M^CNN^
SEN	0.733	0.767	0.133	0.133	0.241	0.310	1	1	0.326	0.473
AUC	0.881	0.913	0.665	0.690	0.535	0.663	1	0.995	0.736	0.753

SEN, sensitivity; AUC, area under the receiver operating characteristic curve; BRCA, breast cancer; GBM, glioblastoma multiforme; CRC, colorectal cancer; THCA, thyroid cancer.

a Each group of samples was a mix of cancer and control samples (n = 666 for groups of T_1_ and n = 260 for groups of T_2_).

b The thresholds of two models were set at 0.9 specificity (M^CAT^: 0.277 for T_1_ and 0.351 for T_2_; M^CNN^: 0.392 for T_1_ and 0.423 for T_2_).

**FIGURE 2:**
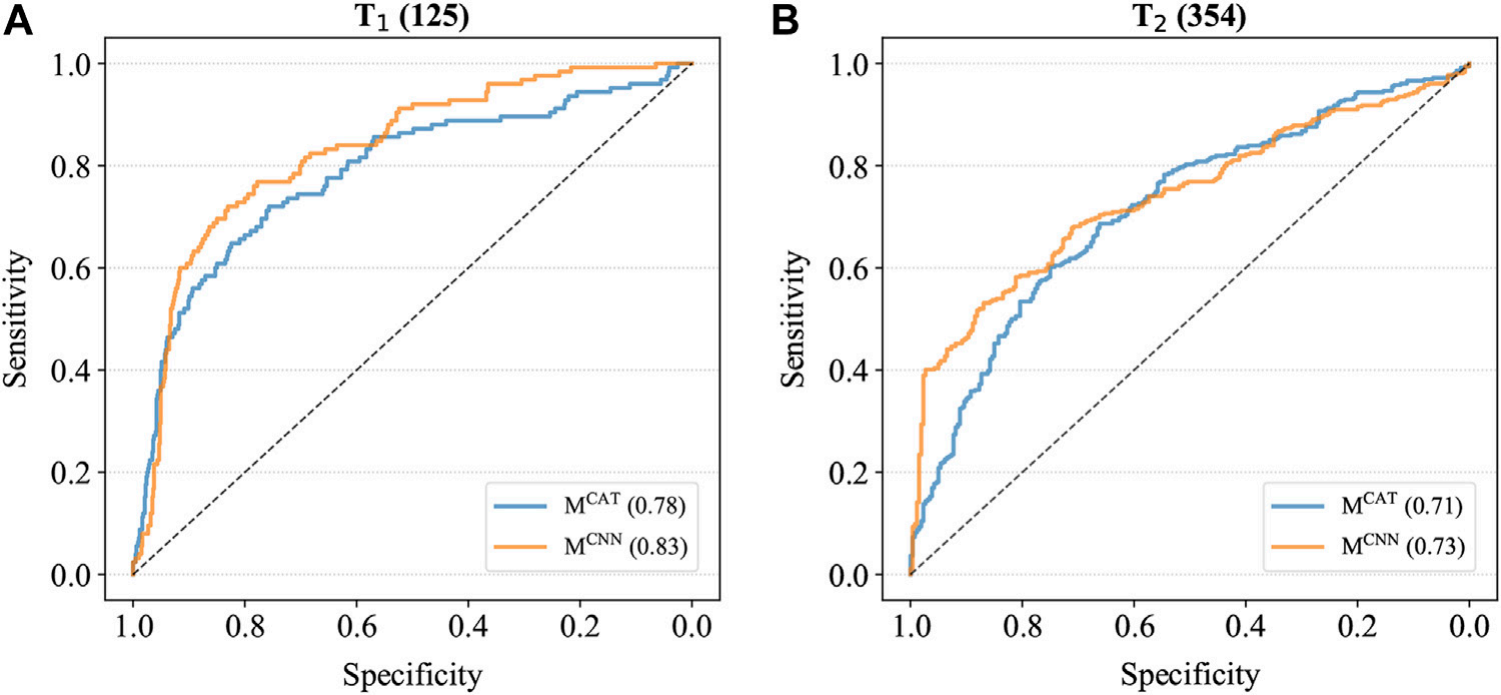
The ROC curves of models on combined cancer samples. **(A)** The ROC curves on T_1_. The AUC of M^CAT^ is 0.78 whereas the AUC of M^CNN^ is 0.83. **(B)** The ROC curves on T_2_. The AUC of M^CAT^ is 0.71 whereas the AUC of M^CNN^ is 0.73.

To further compare the performance of these two models, M^CAT^ and M^CNN^ were also applied to the combined cancer samples of T_1_ and T_2_, which contained all various cancer samples as well as the control samples, and the ROC curves were generated based on the prediction results (Figure 2). As shown in Figure 2, the AUCs of M^CNN^ were higher than those of M^CAT^ on both T_1_ and T_2_ (T1: 0.83 > 0.78; T2: 0.73 > 0.71), indicating that it had the better feature extraction and prediction ability for TCRs.

### Performance Assessment of the Entire DeepLION

In comparison with M^CAT^ and M^CNN^, we expected that the entire DeepLION (M^LION^) as a MIL method was capable of achieving more accurate caTCR prediction after correctly modeling the correlations among TCRs. Because the logistic regression model combined with MIL (denoted as M^LOG^) (Ostmeyer et al., 2019) is a classical MIL method to distinguish tumor tissues from healthy tissues accurately by identifying the cancer-specific motifs in TCRs, we also compared M^LION^ with it.

We trained two models for THCA and lung cancer (denoted as ${\text{M}}_{\text{T}}^{\text{LION}}$ and ${\text{M}}_{\text{L}}^{\text{LION}}$ ) respectively (Table 3). To obtain ${\text{M}}_{\text{T}}^{\text{LION}}$ , we first extracted the top 100 most abundant sequences (*k* = 100) from each sample and encoded them into *l* × 15 TCR matrixes (*d* = 15) by the Beshnova matrix (Beshnova et al., 2020). Then, we trained the model five times independently, in each of which the model was trained with the training set for 700 epochs at a learning rate of 0.001 with the assurance that the model reached convergence. The AUCs of the trained models on the validation set were estimated for model selection, and the model with the highest AUC (0.95) was selected as the final model. ${\text{M}}_{\text{L}}^{\text{LION}}$ (AUC = 0.86) was obtained by the same training procedure. After using the same training sets as M^LION^ to train M^LOG^ with default training parameters, we also obtained ${\text{M}}_{\text{T}}^{\text{LOG}}$ and ${\text{M}}_{\text{L}}^{\text{LOG}}$ for THCA and lung cancer respectively.

**FIGURE 3:**
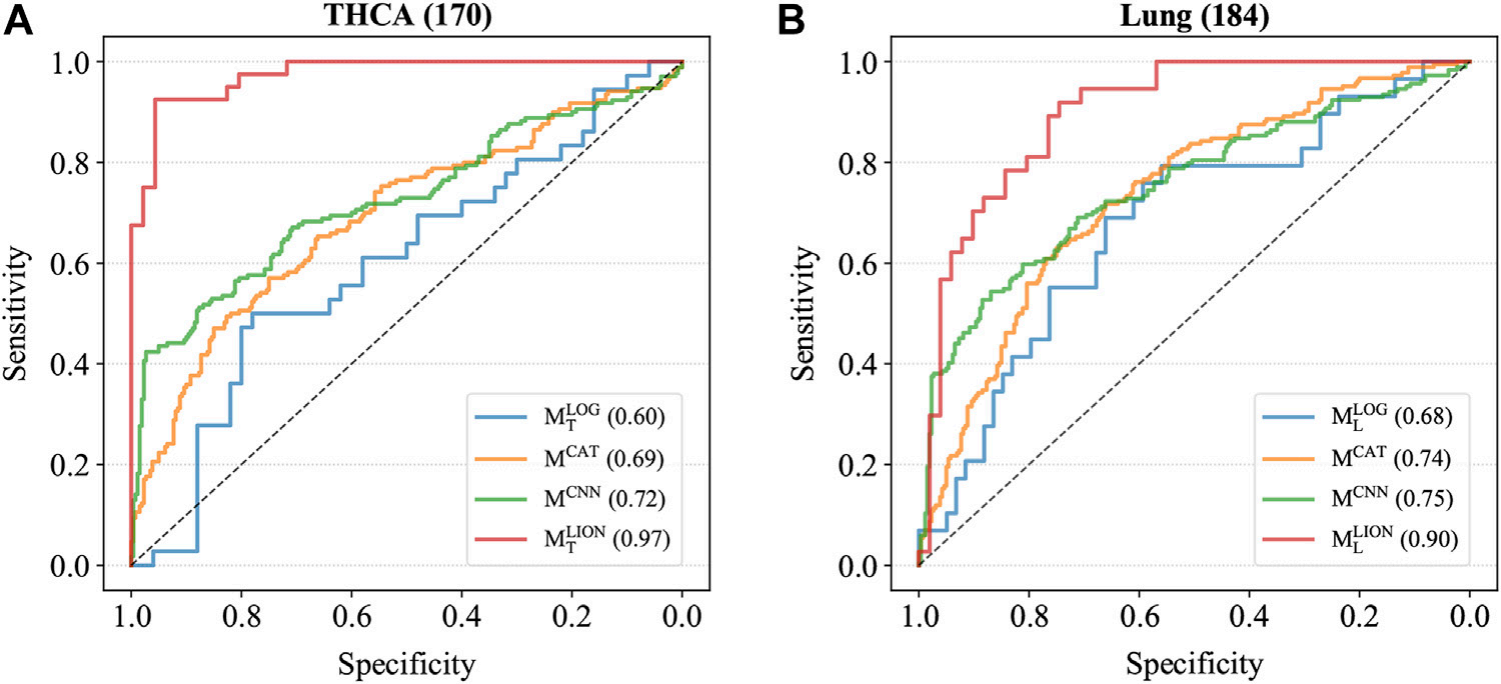
The ROC curves of models on T_2_. **(A)** The ROC curves on THCA samples. The AUCs of M^LOG^, M^CAT^, M^CNN^, M^LION^ are 0.60, 0.69, 0.72 and 0.97. **(B)** The ROC curves on lung cancer samples. The AUCs of M^LOG^, M^CAT^, M^CNN^, M^LION^ are 0.68, 0.74, 0.75 and 0.90.

Four trained models were applied to the corresponding test sets and the accuracies, sensitivities, specificities, and AUCs were calculated based on the sample predictions to evaluate their performances (Table 5). The results of M^CAT^ and M^CNN^ on the samples were also shown in Table 5 for comparison. For a fair comparison, the classification thresholds of M^CAT^ and M^CNN^ for the samples (M^CAT^: 0.336 for THCA and 0.321 for lung cancer; M^CNN^: 0.433 for THCA and 0.419 for lung cancer) were determined by the Youden index (Fluss et al., 2005) enabling the selection of an optimal threshold value for classification, whereas the classification threshold of both M^LOG^ and M^LION^ for any sample is a fixed value (0.5). The ROC curves were generated based on the predicted probabilities of all models on the two samples (Figure 3). The results demonstrated that despite the specificity of M^LION^ being slightly lower than M^CNN^’s on THCA sample, its accuracies, sensitivities, and AUCs were significantly better than the other models on both THCA and lung cancer samples, which indicated that M^LION^ can accurately predict the samples.

**TABLE 5:** The performances of models on THCA and lung cancer samples.

	THCA (170) ^a^				Lung (184)			
	${\text{M}}_{\text{T}}^{\text{LOG}}$ ^b^	M^CAT^	M^CNN^	${\text{M}}_{\text{T}}^{\text{LION}}$	${\text{M}}_{\text{L}}^{\text{LOG}}$	M^CAT^	M^CNN^	${\text{M}}_{\text{L}}^{\text{LION}}$
ACC	0.651	0.693	0.753	0.872	0.659	0.698	0.732	0.818
SEN	0.444	0.488	0.418	0.775	0.552	0.625	0.538	0.730
SPE	0.800	0.827	0.973	0.957	0.712	0.750	0.869	0.882
AUC	0.600	0.692	0.724	0.974	0.680	0.736	0.753	0.899

ACC, accuracy; SEN, sensitivity; SPE, specificity; AUC, area under the receiver operating characteristic curve; THCA, thyroid cancer.

a Each group of samples was a mix of cancer and control samples (n = 260).

b The threshold of both M^LOG^ and M^LION^ was 0.5 for two samples and the thresholds of M^CAT^ and M^CNN^ were set by Youden index (M^CAT^: 0.336 for THCA and 0.321 for lung cancer; M^CNN^: 0.433 for THCA and 0.419 for lung cancer).

## Discussion

In this study, we developed a deep learning method combined with MIL, called DeepLION, to improve the prediction of caTCRs. Compared to some of the current studies that decomposed TCRβ CDR3 sequences into *z*-mers in the data preprocessing, DeepLION was able to extract the features of the cancer-specific motifs with different lengths by the group of various convolution filters and the 1-max pooling operations in the CNN; the MIL part of DeepLION assigned adjusted weights for each TCR after learning the TCR correlations in the prediction process while the existing methods often ignored the correlations among TCRs in the same repertoire. We conducted two experiments on several cohorts of patients from nine cancer types to evaluate the performances of DeepLION. We observed that DeepLION achieved higher prediction accuracies, sensitivities, and AUCs on most of the cohorts than the existing methods, where the AUC reached notably 0.97 and 0.90 for THCA and lung cancer cohorts, respectively.

In section 3.2, with the elaborate design of convolution filters and 1-max pooling operations, M^CNN^ was able to make full use of the information of all TCR sequences in the repertoire (M^CAT^ could only process sequences of length 12–16) and extract the features of motifs of various lengths from TCRs to make more accurate TCR prediction, which resulted in the better performance on the test data (Table 4). However, on some samples, such as glioblastoma multiforme and lung cancer samples in T_1_ and both samples in T_2_, both models performed poorly (low sensitivities and AUCs) (Table 4). This could be due to the ambiguity of the training sequence labels (not all cancer sequences for training were confirmed to be associated with cancer) and the simple definition of the repertoire cancer score, averaging all TCR scores as the repertoire cancer score, which didn’t fully utilize TCR relationships or assign appropriate weights to them. Due to this definition, the models’ classification thresholds were unknown, which is a huge challenge for cancer prediction, despite the high AUCs on samples like melanoma and ovarian cancer samples in T_1_. In our experiments, the thresholds were set based on a fixed specificity to facilitate comparison between models, and the thresholds set at 0.9 specificities for the models were different on T_1_ and T_2_ (M^CAT^: 0.277 for T_1_ and 0.351 for T_2_; M^CNN^: 0.392 for T_1_ and 0.423 for T_2_) (Table 4), indicating that it is difficult for the models to predict precisely for datasets from different sources with a fixed classification threshold. Furthermore, the poor performance of models trained with TCGA data on both Asian samples in T_2_ could be explained by possible differences in TCR repertoires between patients of different races.

In section 3.3, M^LOG^ performed the worst performance on the samples (Table 5), because it applied the inappropriate MIL assumption, using the maximum of TCR score as the repertoire cance score, and used the Atchley matrix (Atchley et al., 2005) to characterize AAs, which contained less biochemical information than the Beshnova matrix (Beshnova et al., 2020), and it couldn’t extract the features of cancer-specific motifs with distinct lengths in the repertoire. Although the Youden index was used to define the classification thresholds for M^CAT^ and M^CNN^, their accuracies and sensitivities on two samples were significantly lower than those of M^LION^ due to the incorrect definition of the repertoire cancer score and the possible differences in the repertoires of patients from different races (Table 5). Different from other methods, M^LION^ used MIL to learn the correlations among TCRs in the repertoire of the patients with the same cancer type, and assign the adjusted weights to each TCR when calculating the cancer scores of the repertoire. Therefore, M^LION^ was capable of predicting the caTCRs and classifying the samples more accurately than existing methods.

When the amounts of training data are small, overfitting is a concern with deep learning models. To reduce overfitting, we simplified the model by using one-layer linear classifiers instead of multi-layer linear classifiers. During the training process, we applied random dropouts at a rate of 40% to each linear classifier and employed early stopping (Yao et al., 2007) to the model. Five-fold cross-validation was performed 10 times on THCA and lung cancer samples separately to assess model generalization (Table 6). Given that K-fold cross-validation produces significantly skewed performance estimates with small sample sizes, whereas nested cross-validation produces robust and unbiased performance estimates regardless of sample size (Wang et al., 2018; Vabalas et al., 2019), we also applied the nested five-fold cross-validation to both THCA and lung cancer samples and repeated it 10 times to ensure the robustness of our evaluation results (Table 6). In comparison to the results of the five-fold cross-validation, some of the metrics in the nested cross-validation results degraded to some extent, but the overall performances of our model were stable, and all the metrics were higher than those of the two existing methods (Table 5), indicating that our model had a high degree of generalizability.

**TABLE 6:** The performances of cross-validations on THCA and lung cancer samples.

	THCA (170) ^a^		Lung (184)	
	K-fold	Nested	K-fold	Nested
ACC	0.843 ± 0.017 ^b^	0.817 ± 0.010	0.786 ± 0.020	0.741 ± 0.010
SEN	0.773 ± 0.025	0.706 ± 0.021	0.697 ± 0.035	0.679 ± 0.026
SPE	0.892 ± 0.035	0.910 ± 0.016	0.848 ± 0.025	0.783 ± 0.019
AUC	0.925 ± 0.010	0.909 ± 0.007	0.841 ± 0.014	0.806 ± 0.011

ACC, accuracy; SEN, sensitivity; SPE, specificity; AUC, area under the receiver operating characteristic curve; THCA, thyroid cancer.

a Each group of samples was a mix of cancer and control samples (n = 260).

b The results show 95% confidence intervals for all the validations (totally 50 validations for each cross-validation).

The results of two cross-validations indicated that our model had higher specificities than sensitivities on both THCA and lung cancer (Table 6). We reasoned that this is likely because some caTCRs have sequence similarities with non-cancer TCRs, but their antigenic specificities differ due to their different spatial structures; there are many more non-cancer TCRs than caTCRs in a cancer-associated repertoire, which also brings additional difficulty to identifying the caTCRs based on the limited TCR-seq data. Furthermore, our model is rigorous in determining cancer samples and makes negative judgments on ambiguous samples. As a result, the model is more prone to making mistakes when it comes to predicting cancer samples. Likewise, the existing methods also had lower sensitivities than specificities (Table 5). But DeepLION outperformed them in cancer sample prediction due to the unique architecture of CNN and the MIL part.

DeepLION has few adjustable hyperparameters except for the number of TCRs extracted from one repertoire *k* and the learning rate, which is easily applied. The computational complexity of the model is lower when *k* is smaller, but the information of TCRs used by the model is less, which could reduce the model performance. Thus, *k* should be as small as possible while ensuring satisfactory model performance. Because we observed that the model performance was unsatisfactory when *k* < 100 through experiments, we finally set *k* to 100 in our experiments. In addition, the abundance of TCRs affects the predictions of the model. We discovered that the model performance deteriorated if we randomly extracted the 100 TCRs instead of the top 100 TCRs. Because the learning rate is usually set to 0.001, we used this value as well.

In future work, we will apply DeepLION to other cancer types. And we plan to improve our method to make it able to extract the shared correlations among TCRs of patients with various cancers so that the trained model can be applied to detect various cancers accurately. In this method, we will utilize TCRα sequences as well as TCRβ sequences for analysis and explore a better encoding operation to characterize TCRs.

## Conclusion

DeepLION introduces a deep MIL framework to consider the various length of the cancer-associated motifs and the correlations among TCRs, achieving a higher prediction accuracy in cancer detection from TCR repertoire data than the current state-of-the-art methods. Thus, DeepLION has the potential to support cancer detection from TCR repertoire data.

## Data Availability

The source codes and the software tool, DeepLION, is available on GitHub, at https://github.com/Bioinformatics7181/DeepLION, for academic usage only. The publicly available TCR-sequencing data for this study can be found in Adaptive Biotechnologies immuneACCESS online database, at https://clients.adaptivebiotech.com/immuneaccess. The thyroid cancer TCR-sequencing samples were derived from Lan et al. (Lan et al., 2020), which can be found in NCBI, at https://www.ncbi.nlm.nih.gov/bioproject/PRJNA642967, whereas the lung cancer TCR-sequencing samples were derived from Li et al. (Li et al., 2021). The training data of DeepCAT and the trained DeepCAT model can be found on Github, at https://github.com/s175573/DeepCAT.

## Abbreviations

AA, Amino acid; ACC, Accuracy; AUC, Area under the receiver operating characteristic curve; AVG, Average; BRCA, Breast cancer; caTCR, Cancer-associated T cell receptor; CDR3, Comple-mentarity determining region 3; CMV, Cytomegalovirus; CNN, Convolutional neural network; CRC, Colorectal cancer; GBM, Glioblastoma multiforme; IA, Adaptive Biotechnologies immuneACCESS online database; IR-seq, immune repertoire sequencing; MHC, Major histocompatibility complex; MIL, Multi-instance learning; PBMC, Peripheral blood mononuclear cell; PCA, principal component analysis; ReLU, Rectified linear unit; RNA-seq, Ribonucleic acid-sequencing; ROC, Receiver operating characteristic; SEN, Sensitivity; SPE, Specificity; TCGA, The Cancer Genome Atlas; TCR, T cell receptor; TCR-seq, T cell receptor-sequencing; THCA, Thyroid cancer; TIL, Tumor-infiltrating T lymphocyte.
